# The immunoregulatory effects of scoparone on immune-mediated inflammatory diseases

**DOI:** 10.3389/fimmu.2025.1518886

**Published:** 2025-01-31

**Authors:** Feifei Qiu, Jingru Lin, Xiaofei Huang, Bin Yang, Weihui Lu, Zhenhua Dai

**Affiliations:** ^1^ Section of Immunology, the Second Affiliated Hospital of Guangzhou University of Chinese Medicine, Guangzhou, Guangdong, China; ^2^ Department of Cardiovascular Sciences, College of Life Sciences University of Leicester, Leicester, United Kingdom; ^3^ State Key Laboratory of Dampness Syndrome of Chinese Medicine, the Second Affiliated Hospital of Guangzhou University of Chinese Medicine, Guangzhou, Guangdong, China

**Keywords:** inflammatory disease, immunoregulation, liver disease, scoparone, signaling pathway

## Abstract

Scoparone (SCO), also known as 6,7-Dimethoxycoumarin, is a naturally occurring bioactive ingredient originally derived from Chinese herb Artemisiae Scopariae Herba (Yin-Chen-Hao). Previous studies have shown that it is effective in treating some of the liver diseases. Beyond its hepatoprotective effects, an expanding body of research has underscored the immunoregulatory properties of SCO, indicating its potential therapeutic benefits for autoimmune and other inflammatory diseases. Over the past decade, significant advances have been made in understanding the mechanistic insights into its effects on immune-mediated diseases as well as liver diseases. SCO has an impact on various immune cells, including mast cells, monocytes, macrophages, neutrophils and T cells, and affects a broad range of intracellular signaling pathways, including TLR4/Myd88/NFκB, TGFβR/Smad3 and JNK/Sab/SHP-1 etc. Therefore, this review not only summarizes the immunomodulatory and therapeutic effects of SCO on immune-based inflammatory diseases (IMIDs), such as inflammatory bowel disease, osteoarthritis, allergic rhinitis, acute lung injury, type 1 diabetes and neuroinflammatory diseases etc., but also provides a comprehensive summary of its therapeutic effects on hepatic diseases, including non-alcoholic steatohepatitis, fulminant hepatic failure and hepatic fibrosis. In this review, we also include the broad impacts of SCO on intracellular signaling pathways, such as TLR4/Myd88/NFκB, TGFβR/Smad3, Nrf2/P38, JAK2/STAT3 and JNK/Sab/SHP-1 etc. Further researches on SCO may help understand its in-depth mechanisms of action and pave the way for the development of novel drugs to prevent and treat various immune-mediated inflammatory disorders as well as hepatic diseases, thereby significantly advancing its innovations and pharmaceutical applications.

## Introduction

Scoparone (6,7-Dimethoxycoumarin, SCO), a naturally occurring bioactive ingredient originally derived from Chinese herb Artemisiae Scopariae Herba (Yin-Chen-Hao), possesses the diverse pharmacological properties, including anti-inflammatory, antioxidant and anti-cholestatic effects. Yin-Chen-Hao has been commonly used in the treatment of liver and bile disorders, such as acute jaundice, hepatitis and cholestasis disorders (1). Notably, SCO serves as a principal active component in the traditional formulation of Yin-Chen-Hao (YCH) and Yinzhihuang decoctions (YZHDs), which have been clinically administered to treat liver and cholestasis ailments (2, 3). Recent studies have made significant progresses in understanding the mechanisms by which SCO, or in combination with other pharmaceutical ingredients, exerts its anti-inflammatory effects.

Immune-mediated inflammatory diseases (IMIDs) include a broad condition of organ/tissue inflammation characterized by dysregulated immune responses, resulting in inflammation and the subsequent damage to target organs (4, 5). Previous studies have reported the effects of SCO on IMIDs, which are mostly autoimmune and allergic diseases, such as inflammatory bowel disease, osteoarthritis, allergic rhinitis, type 1 diabetes and neuroinflammatory diseases etc. Researchers have also demonstrated its therapeutic effects on hepatic diseases, including non-alcoholic steatohepatitis, fulminant hepatic failure and hepatic fibrosis (6). In this review, we have summarized recent studies exploring the immunomodulatory and anti-inflammatory effects of SCO on immune-related inflammatory and liver diseases, as well as the cellular and molecular mechanisms underlying its immunoregulatory and anti-inflammatory effects (**Table 1**). We also provided potential research prospects for the treatment of various IMIDs using SCO, thus helping lay the foundation for its clinical trials to treat various inflammatory diseases, especially autoimmune diseases.

**Table 1:** Effects of scoparone on immune-mediated inflammatory diseases and hepatic diseases.

	Disease	Cell type or tissue	Effects	Signaling Pathway	Ref
**Immune-mediated inflammatory** **diseases**	Inflammatory bowel disease	The colonic segments	↓Oxidative stress, glutathione consumption,AP; ↑GSH		(46)
	Osteoarthritis	Chondrocyte	↓NO,MMP-3,MMP-13, ADAMTS-4, ADAMTS-5 and PGE2, iNOS,COX-2	↓PI3K/Akt/NF-κB pathway	(3)
	Acute lung injury	Neutrophils, macrophages	↓CXCL1, CXCL2,CCL2, TNF-α, IL-6, IL-1β	↓TLR4/NF-κB pathway	(19)
	Allergic rhinitis	Th1, Th2	↓IgE, IL-4, IL-5, TLR4, p65, TLR4; ↑IFN-γ	↓TLR4/NF-κB pathway	(55)
			↓IgE, IL-4, IL-5; ↑IFN-γ		(25)
	Diabetes	β-cell	↓nuclear translocation of NF-κB; ↓β-cell damage, insulin secretion; ↓iNOS, IL-1β, IFN-γ and NO	↓NF-κB pathway	(57)
	Alzheimer	Microglia	↓TLR4, MyD88, TRAF-6, TAK-1 and NLRP3; ↑microglia transition towards M2 subtype	↓TLR4/MyD88/TRAF-6/TAK-1/NF-κB pathway	(63)
	Epilepsy	Hippocampus	↓GFAP, Iba1, CXCL1, IL-1β, TNF-α, MCP-1, IL-6, HIF-1α and HMGB1; ↓TLR4, MyD88, p-IκBα, p-NF-κB; ↓PI3K, p-AKT and p-GSK-3β; ↓Casapse-3 cleaved; ↓astrocyte activity, inflammation and apoptosis	↓TLR4/NF-κB and PI3K/AKT pathway	(64)
**Hepatic diseases**	Non-alcoholic steatohepatitis	Hepatocytes	↓P-JNK/JNK and P-SHP-1/SHP-1;↑P-Src/Src; ↓mRNA expression of IL-1β and TNF-α; ↓Cleaved PARP;↓oxidative stress levels, ROS and lipid peroxide 4HNE	↓JNK/Sab pathway	(30)
		Liver macrophages	↓cleaved caspase-3;↓mRNA levels of MCP-1, TNF-α, IL-6, IL-1β and iNOS; ↑IL-10, Arg1 and IL-1RA; ↓α-SMA, ICAM1,COL1A1 TGF-β1 and CTGF	↓TLR-4/NF-κB pathway	(18)
		Hepatocytes	↓mRNA levels of TNF-α, IL-6, IL-1β; ↓oxidative stress levels	↑AMPK pathway	(31)
		Hepatocytes	↓mRNA levels of TNF-α, IL-6, IL-1β, Rela; ↑Klf10	↑PPARα pathway	(32)
		Hepatocytes	↓liver levels of TNF-α, IL-6, IL-1β, NLRP3 inflammasome, ASC; ↑liver levels of SOD, CAT	↓NLRP3/IL-1β pathway	(33)
	Fulminant hepatic failure	Liver tissues	↓p-p38, p-ERK1/2, p-JNK, p65; ↓TNF-α, IL-6, TRIF, IFN-β; ↓IRF3 phosphorylation	↓TLR-4/NF-κB pathway	(37)
			↑zinc finger protein 407, prothrombin, transthyretin; ↓alpha-1-antitrypsin, haptoglobin		(38)
	Hepatic fibrosis	Hepatic stellate cells	↓TGF-β1, NOXs, ROS and p-Smad3; ↓α-SMA, collagen I and collagen III	↓TGF-β/Smad pathway	(41)

Arrows upward denote “increasing”, while arrows downward indicate “decreasing or suppressing” in this table.

## The direct effects of SCO on immune cells

**Figure 1:**
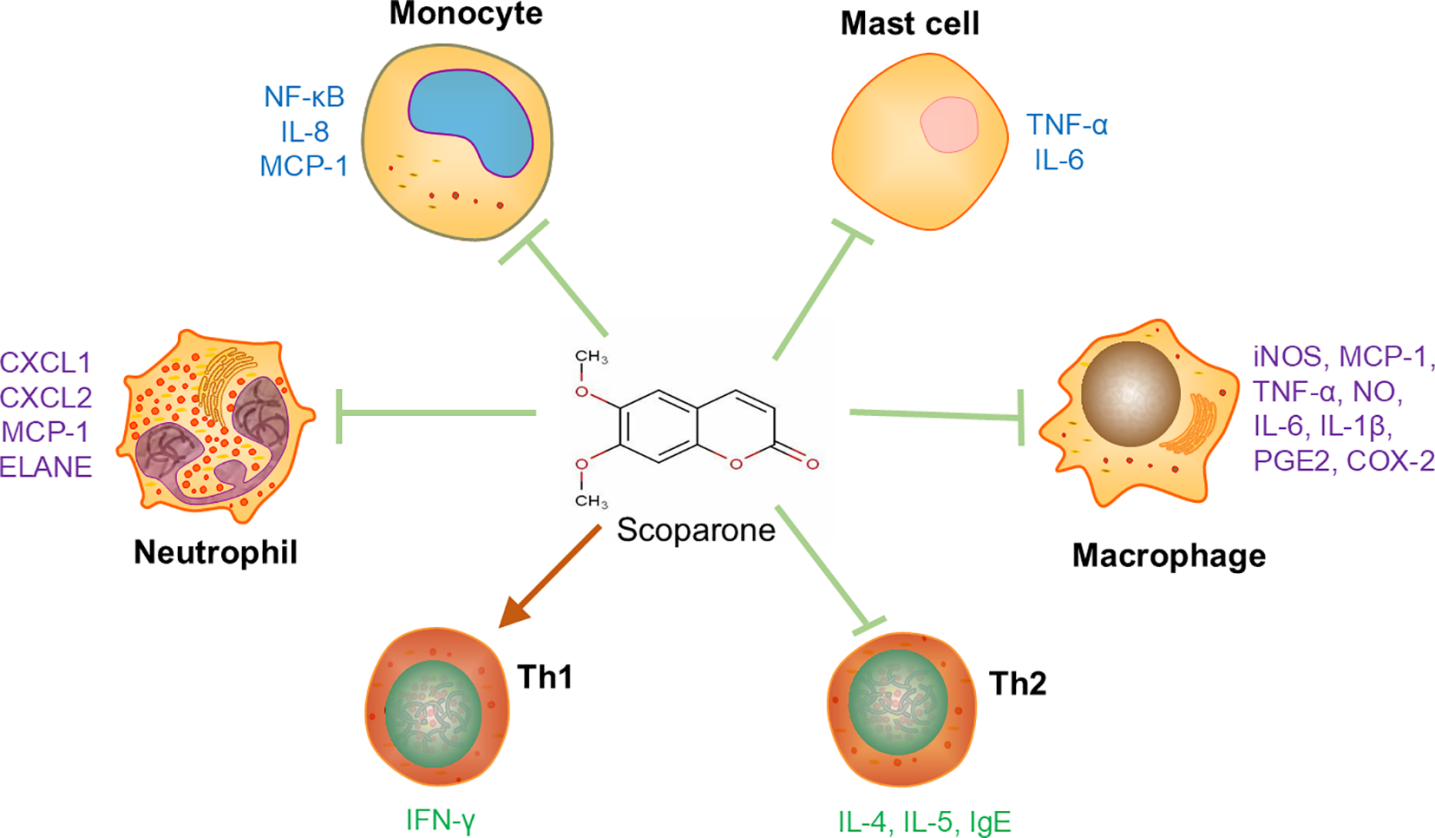
The effects of scoparone on adaptive and innate immune cells. Scoparone can regulate the cytokine expression in T helper cells, macrophages, neutrophils, mast cells and monocytes. Arrows “↓” mean “enhancing” while “⊥” means “inhibiting”. Th1, T helper cell 1; Th2, T helper cell 2; CXCL1, C-X-C motif chemokine ligand 1; CXCL2, C-X-C motif chemokine ligand 2; COX-2, cyclooxygenase 2; ELANE, elastase, neutrophil-expressed; PGE2, prostaglandin E2.

Immune cells play important roles in the development and progression of immune-mediated inflammatory diseases (IMIDs). SCO alleviates the deterioration of IMIDs by regulating immune responses as well as the expression of numerous cytokines/chemokines in various immune cells (**Figure 1**).

### Mast cells

Mast cells play a key role in anaphylaxis by releasing a variety of pro-inflammatory mediators and cytokines. It was found that activated mast cells produced tumor necrosis factor (TNF-α) and interleukin (IL)-6, which played a central role in triggering and maintaining allergic inflammation (7). Previous studies also demonstrated that SCO inhibited the expression of TNF-α and IL-6 proteins and the activation of p38 MAPK and NF-kB signaling pathways through prohibiting the uptake of calcium by mast cells, thereby alleviating IgE-mediated mast cell hypersensitivity (8). Thus, SCO may be used to treat allergic diseases.

### Mononuclear cells

Mononuclear cells normally maintain the inflammatory balance and immune tolerance in a physiological condition (9). Previous results showed that SCO inhibited the proliferation of mononuclear cells in a dose-dependent manner in mixed lymphocyte responses (10, 11). SCO antagonized the effects of the diabetogenic drug alloxan on mononuclear cells and increased the levels of PGE2, PGF2α, leukotriene B4 and 2,3-dinor-thromboxane B2 in phytohemagglutinin (PHA)-stimulated monocyte culture (10). Moreover, SCO inhibited the response of mononuclear cells to PHA, mixed lymphocytes, mitogen and alloantigen, while it played a role in relaxing blood vessels and suppressing immunity (11). Another study also reported that SCO suppressed NF-κB activation in U937 human monocytes activated by phorbol 12-myristate 13-acetate (PMA) and reduced PMA-induced toxicity and release of IL-8 and MCP-1 proteins (12).

### Macrophages

Macrophages process antigens and carry them to the lymph nodes, while also secreting pro-inflammatory factors (13). Yeh et al. found that SCO not only inhibited M1 macrophage markers, such as iNOS, IL-6 and CCL2, but also significantly reduced the protein level of TNF-α in LPS-treated macrophages (14). In addition, they reported that SCO significantly increased the gene expression of M2 macrophage markers and the protein level of Arg1, thus promoting the differentiation of macrophages towards anti-inflammatory M2 phenotype. Similarly, Liu et al. demonstrated that SCO suppressed macrophage autophagy and M1 polarization (15). Previous studies also showed that SCO inhibited the activation of NLRP3 inflammasome in LPS-induced murine macrophages and mouse models of bacterial enteritis and septic shock, and suppressed the production of TNFα, IL-6, NO and PGE2 in IFN-gamma/LPS stimulated RAW 264.7 cells (16, 17). Liu et al. proved that SCO mitigated macrophage responses induced by NASH and LPS via blocking the TLR-4/NF-κB signaling pathway (18). Invasive liver macrophages led to chronic liver injury and fibrosis through HSC transdifferentiation and proliferation, while SCO inhibited HSC activation, downregulated macrophage infiltration, suppressed the secretion of NO, PGE2, iNOS and COX-2 in RAW 264.7 cells, and reduced the production of TNF-α, IL-1β and IL-6 in RAW264.7 cells stimulated by LPS (17). Similarly, Niu et al. found that SCO inhibited NF-κB activation and TLR4 expression as well as the production of TNF-α, IL-6 and IL-1β in LPS-induced alveolar macrophages *in vitro* (19). Thus, SCO suppressed the expression of pro-inflammatory cytokines in macrophages and promoted their polarization towards M2 phenotype, suggesting that SCO may be effective in treating macrophage-mediated inflammatory diseases.

### Neutrophils

Neutrophils play a pivotal role in the innate immune responsiveness and mainly exert pro-inflammatory effects, causing severe tissue inflammation (20). Experiments by Niu et al. demonstrated that SCO significantly diminished neutrophil numbers and downregulated the expression of CXCL1, CXCL2 and CCL2, thereby mitigating inflammation (19). Studies by Chan et al. showed that SCO (6,7-dimethoxycoumarin) exhibited significant inhibition of superoxide anion generation and the release of elastase by human neutrophils *in vitro* (21). These findings indicate that SCO can exert anti-inflammatory effects.

### T cells

T cells are a core component of adaptive immunity, mainly including type 1 helper T cells (Th1), type 2 helper T cells (Th2) and Th17 cells classified under CD4^+^ T cells (22). The immunological basis of allergic rhinitis (AR) was closely related to the imbalance between Th1 and Th2 cells (23). Th2 cytokines IL-4 and IL-5 were involved in AR allergic inflammation (24). SCO inhibited the synthesis of IgE, upregulated the level of Th1 cytokines in serum, reduced the level of Th2 cytokines, and restored the balance between Th1 and Th2 cells, thus improving the symptoms of allergic rhinitis in rats (25).

## Effects of SCO on inflammatory liver diseases and other IMIDs

SCO has been shown to treat various hepatic diseases in animal models (6), including immune-associated inflammatory liver diseases. It also has been used to treat many of the other IMIDs in pre-clinical studies. In fact, SCO has demonstrated the efficacy in treating various IMIDs, including the intestinal disorders, respiratory ailments and osteoarthritis etc. IMIDs encompass a spectrum of conditions characterized by dysregulated immune responses, leading to inflammation and subsequent damage to target organs (4, 5). Some of the prevalent IMIDs are classified as autoimmune diseases, in which the immune system mistakenly targets and attacks the self organ/tissue. These conditions are often accompanied with a range of comorbidities, including cardiovascular diseases, liver diseases, metabolic disorders, skeletal abnormalities and cognitive impairments (26, 27).

### Non-alcoholic steatohepatitis

Non-alcoholic steatohepatitis(NASH), a progressive form of non-alcoholic fatty liver disease and a prevalent chronic liver condition, is characterized by inflammation without or with fibrosis in addition to the hepatic steatosis (28). Previous studies have shown that NASH is caused by lipotoxic liver injury, in which excess lipid accumulation promotes insulin resistance, oxidative stress, mitochondrial dysfunction and endoplasmic reticulum stress, leading to apoptosis, inflammation and liver tissue fibrosis (29). Study by Jiang et al. showed that the activation of JNK/Sab signaling pathway induced by palmitic acid was blocked by SCO treatment, with a decrease in the blood lipid and aminotransferase, therefore improving liver histopathological conditions via restoration of mitochondrial function and reversal of hepatic steatosis in mice (30). Liu et al. found that SCO also improved liver steatosis, apoptosis, inflammation and fibrosis in a mouse model of MCD diet-induced NASH. Additionally, they highlighted capacity of SCO to modulate immune responses in LPS-induced RAW264.7 macrophages through the inhibition of TLR-4/NF-κB signaling pathway (18). Wei et al. revealed that SCO could reduce murine liver injury, oxidative stress and inflammation by suppressing lipid accumulation and improving alcohol metabolism (31). SCO also was shown to decrease the expression of TNF-α, IL-6, IL-1β and Rela, increase the expression of krueppel-like factor 10 (Klf10), and attenuate lipid metabolism dysfunction and inflammation by activating the peroxisome proliferator-activated receptor α (PPARα) signaling pathway (32). Zhao et al. showed that SCO alone reduced the expression of apoptosis-associated speck-like protein containing a CARD (ASC), IL-1β, TNF-α and IL-6, inhibited the activation of NLRP3 inflammasome in the liver, and increased the levels of the antioxidants CAT and SOD, thus ameliorating liver inflammation (33). Finally, scoparone treatment improved glycerophospholipid metabolism and liver histopathology in a murine NASH model (34). Collectively, those findings underscored the potential protective effects of SCO on NASH and its hepatic pathology.

### Fulminant hepatic failure

Fulminant hepatic failure (FHF) is a severe clinical syndrome with extensive liver cell damage. Toxic hepatitis and acute viral hepatitis represent the most prevalent etiologies of FHF (35). Proinflammatory cytokines and chemokines promote oxidative stress in the damage caused by toxic substances, leading to the massive infiltration of proinflammatory cells and eventually the formation of severe hepatitis (36). Kang et al. demonstrated that the pathogenesis of FHF was related to the upregulation of MyD88 and TRF-dependent signaling pathways in the TLR system. Their results showed that pretreatment with SCO inhibited the expression of TLR4/Myd88 and suppressed the phosphorylation of p38, ERK1/2 and cJun N-terminal kinase (JNK) as well as the activation of NF-κB in mice with D-galactosamine(D-GalN)/LPS-induced FHF (37). Zhang et al. used STRING analyses to map the protein interaction networks and found that the liver protective effects of SCO on acute liver injury in rats were associated with the expression of six proteins, including Ig kappa chain C, zinc finger protein 407, prothrombin, haptoglobin, alpha-1-antitrypsin and transthyretin, thus providing new insights into the mechanisms responsible for its liver protection (38).

### Hepatic fibrosis

Hepatic fibrosis is a chronic liver disease characterized by excessive production and deposition of extracellular matrix (ECM) in the liver. The disease mainly develops from chronic liver inflammation caused by viral hepatitis, alcoholism, metabolic drugs and steatohepatitis, etc (39). The activation of hepatic stellate cells (HSC) is a main mechanism underlying the chronic hepatic fibrosis (40). Experiments by Liu et al. demonstrated that SCO significantly reduced the expression of NADPH oxidase (NOX), production of ROS, and Smad3 phosphorylation in TGF-β1-stimulated HSC T6 cells *in vitro* (41), suggesting that SCO likely alleviates liver fibrosis, which is usually caused by chronic inflammation.

### Inflammatory bowel disease

Inflammatory bowel disease (IBD), including Crohn’s disease and ulcerative colitis, represents a chronic, recurrent and immune-mediated condition that inflicts damage on the gastrointestinal tracts and significantly impairs patients’ quality of life (42, 43). Oxidative stress induced by free radicals and reactive oxygen species is strongly implicated in the pathogenesis of IBD, which is characterized by diminished endogenous antioxidants and heightened oxidative stress biomarkers (44, 45). Witaicenis et al. previously reported that administration of SCO significantly reduced the incidence of diarrhea, injury score and colon weight in a mouse model of trinitrobenzene sulfonic acid (TNBS)-induced colitis (46). Besides, they also observed that SCO decreased the activity of alkaline phosphatase (AP), a sensitive marker of intestinal inflammation (46), and carbon tetrachloride-induced oxidative stress but enhanced the expression of gamma-glutamyl-cysteinyl-glycine (GSH), which acted as an endogenous reactive oxygen scavenger (47). These findings imply that the protective effects of SCO on intestinal inflammation are associated with its antioxidant property.

### Osteoarthritis

Osteoarthritis (OA) is a progressive joint ailment characterized by joint swelling, pain and functional impairment due to the damage to cartilage, bone and the synovial cavity (48). Previous investigations have revealed that synovial inflammation in OA involves the infiltration of macrophages and T cells, with increased levels of Th1, Th9 and Th17 cells in OA joint fluid (49). A pro-inflammatory cytokine interleukin-1β (IL-1β) was implicated in OA pathogenesis, and capable of inducing chondrocyte senescence (50). Treatment with SCO weakened the effects of IL-1β on chondrocyte viability by decreasing the expression of NO, PGE2, MMP-3, MMP-13, ADAMTS-4, ADAMTS-5, iNOS and COX-2 in a dose-dependent manner (3). Meanwhile, SCO also interfered with the development of OA by regulating the PI3K/Akt/NF-κB signaling pathway (3). These findings indicate that SCO is potentially a therapeutic agent for OA management and treatment.

### Acute lung injury

Acute lung injury (ALI) is an acute inflammatory disease that commonly occurs in clinical settings with a high mortality rate, posing a significant threat to patients’ life. It was reported that LPS induced the production of pro-inflammatory cytokines TNF-α, IL-6, IL-1β and IFNγ etc. (51), thereby accelerating the pathophysiological process of endotoxin-induced ALI. Previous studies showed that LPS, as a key risk factor for ALI, stimulated alveolar macrophages to activate Toll-like receptor 4 (TLR4) signaling (52), while LPS could also upregulate the expression of chemokines CXCL1, CXCL2 and CCL2 on the margins of macrophages and neutrophils in the lung (53).

Study performed by Niu et al. demonstrated that SCO exerted a protective effect on LPS-induced ALI. They used a mouse model of acute lung injury induced by LPS through nasal gavage and found that treatment with SCO inhibited the accumulation of pulmonary neutrophils and macrophages and suppressed myeloperoxidase activity and expression of CXCL1, CXCL2, CCL2, TNF-α, IL-6 and IL-1β *in vivo*, resulting in the mitigation of pulmonary edema and damage. Additionally, SCO was found to inhibit TLR4-mediated NF-κB signaling pathway and the expression of TLR4, TNF-α, IL-6 and IL-1β in LPS-stimulated alveolar macrophages, underscoring its efficacy in alleviating LPS-induced ALI (19). Thus, SCO may be implicated for the treatment of lung diseases that cause an ALI condition.

### Allergic rhinitis

Allergic rhinitis (AR) is a type 1 allergic disease mediated by IgE (54). Experiments performed by Yuan et al. showed that SCO ameliorated AR in rats by regulating the balance of Th1 and Th2 immune responses via inhibiting TLR4/NF-κB signaling pathway. They reported that SCO treatment led to a reduction in rhinitis symptom scores and decrease in serum levels of IgE, IL-4 and IL-5, with an increase in IFN-γ level (55). Similar results were observed by Cheng et al. showing that the pro-inflammatory cells of mice with AR were significantly reduced after SCO treatment, with the mucosal structure returning to normal. Compared with AR group, the serum level of IFN-γ was increased, while that of IL-4, IL-5 or IgE was significantly decreased in SCO treatment group (25). Therefore, these results suggest that SCO can inhibit or eliminate symptoms of AR by improving the imbalance of Th1/Th2 immune responses.

### Type 1 diabetes

Type 1 diabetes, commonly referred to as autoimmune diabetes, arises from the autoimmunity-mediated destruction of insulin-producing β cells within the pancreas and is influenced by genetic predisposition and potential environmental triggers (56). Cytokines produced by immune cells infiltrating pancreatic islets serve as pivotal mediators in the destruction of β cells, leading to insulin-dependent diabetes mellitus. Kim et al. demonstrated, for the first time, that SCO treatment not only protected rat insulinoma cells stimulated by IL-1β and IFN-γ, but also maintained the secretion of insulin by islets stimulated by glucose, while SCO also inhibited NF-κB nuclear translocation, thereby suppressing the expression of cytokine-induced iNOS (57). These findings have underscored the therapeutic potential of SCO for halting the progression of type 1 diabetes.

### Neuroinflammatory diseases

Neuroinflammation is an inflammatory reaction in the brain or spinal cord, which is mediated by cytokines, chemokines, reactive oxygen species, etc. (58). Activation of microglia leads to elevated levels of the neurotoxic and pro-inflammatory mediators, resulting in severe damage to brain cells and occurrence of various neuroinflammatory diseases, including Alzheimer’s disease (AD) and epilepsy (59). Thus, modulation of microglial activation emerges as a pivotal strategy for averting diverse neuroinflammatory diseases.

SCO is gaining prominence in neurotherapeutics owing to its low toxicity and ability to inhibit microglial activation. Santibanez et al. employed HPLC-DAD-UV bioassays to elucidate the temporal concentration profile of each coumarin in a LPS-induced neuroinflammation model. They revealed that SCO maintained elevated tissue concentrations compared to other compounds, exhibiting notable bioavailability in both brain tissue and plasma. This finding demonstrated the capacity of SCO to traverse the blood-brain barrier, indicating its potential advantage in treating various neuroinflammatory diseases (60).

Recent studies have also unveiled some mechanisms underlying the pharmacological effects of SCO. Cho and colleagues found that SCO inhibited neuroinflammation by reducing ERK and the TRIF-dependent signaling molecule IRF3, rather than affecting the activation of NF-κB and MAPK in LPS-stimulated BV-2 microglia (61). Neuroinflammation mediated by microglia activation is a key factor in the onset of Alzheimer’s disease (62). As reported by Ibrahim et al., SCO promoted the polarization of microglia toward an M2 subtype by shutting down the TLR4/MyD88/TRAF-6/TAK-1/NF-κB axis and inhibiting the NLRP3 pathway, and effectively mitigated ovariectomy/D-galactose(OVX/D-Gal)-induced neuroinflammation and neuronal degeneration, leading to a reduction in neuroinflammation and neurodegeneration in OVX/D-Gal models of Alzheimer’s disease (63). Besides, SCO significantly reduced the expression of GFAP and Iba1 in the cortex of epileptic mice and inhibited the expression of CXCL-1, IL-1β, TNF-α, MCP-1, IL-6, HIF-1 and HMGB1, thereby protecting mice from pilocarpine-induced seizures by inhibiting Casapse-3 fragmentation, activation of astrocytes and microglia, inflammation and cell apoptosis through the TLR4/NF-κB pathway (64). These findings underscored the potential of SCO as an anti-neuroinflammatory agent and offered promising avenues for drug development in various neuroinflammatory diseases.

## The mechanisms underlying the effects of SCO: its impacts on intracellular signaling pathways

**Figure 2:**
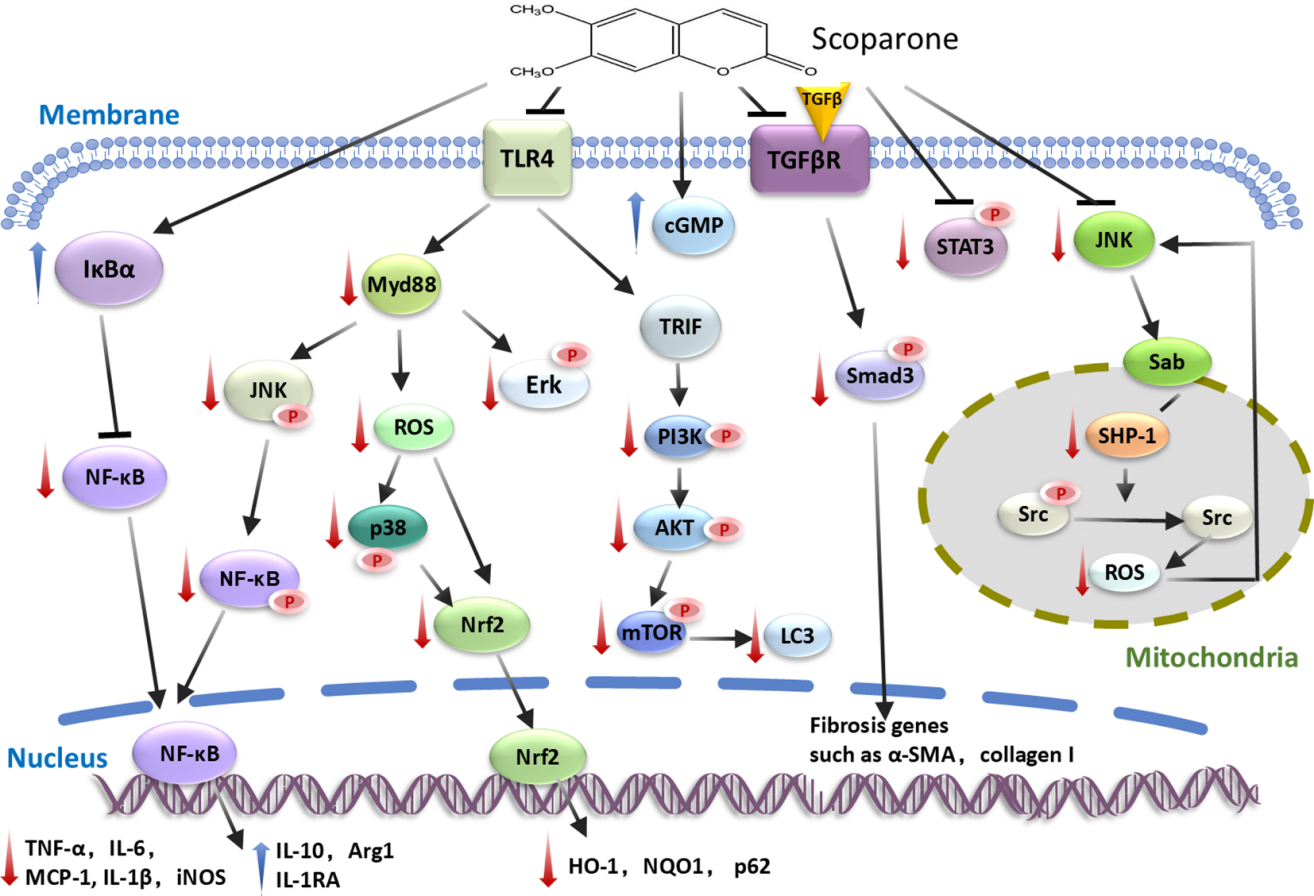
Signaling pathways regulated by scoparone. By interfering with TLR/NF-κB, PI3K-Akt, Nrf2, JNK/Sab, TGF-β/Smad, Nitric oxide (NO)-cGMP and JAK2-STAT3 signaling pathways, scoparone downregulates pro-inflammatory genes and upregulates anti-inflammatory and antioxidant genes. The downward red arrows indicate inhibition, while the upward blue arrows denote stimulation/increase. α-SMA, actin alpha 2; cGMP, cyclic guanosine monophosphate; HO-1, oxygenase 1; iNOS, inducible nitric oxide synthase; MCP-1, monocyte chemoattractant protein-1; NQO1, NADPH:quinone oxidoreductase 1; Nrf2, NFE2 like bZIP transcription factor 2; ROS, reactive oxygen species; SHP-1, SH2 domain-containing protein tyrosine phosphatase 1; Smad3, SMAD family member 3; Sab, SH3-domain binding protein 5; STAT3, signal transducer and activator of transcription 3; TLR4, Toll like receptor 4.

SCO regulates immune homeostasis and ameliorates inflammatory diseases through acting on the complex signaling networks. SCO modulates cellular responsiveness by altering many of the intracellular signaling pathways, such as TLR/NF-κB, PI3K-Akt, Nrf2, JNK/Sab, TGF−β/Smad, Nitric oxide (NO)-cGMP and JAK2-STAT3 signaling axes (**Figure 2**).

### TLR/NF-κB

Nuclear factor-kappa B (NF-κB) signaling plays a vital role in both immunity and inflammation. It regulates the expression of various proinflammatory genes and serves as a critical mediator for inflammatory responses (65). The activation of NF-κB induces many of the pro-inflammatory mediators and molecules, leading to inflammatory responses caused by the activation of various immune cells (66). Thus, NF-κB is tightly regulated to maintain the immunological balance. Niu et al. used a murine model of acute lung injury induced by LPS via nasal gavage and found that SCO inhibited TLR4-mediated NF-κB activation and the production of proinflammatory cytokines, such as TNF-α, IL-1β and IL-6, thereby suppressing inflammation (19). Another study demonstrated that SCO blocked LPS-stimulated increases in the levels of TLR4/MyD88 proteins and downstream phosphorylated NF-κB activity in RAW264.7 cells (18). Kang et al. revealed that SCO attenuated IgE-mediated allergic and inflammatory responses in mast cells by inhibiting the overexpression of TLR4 and blocking NF-κB activation (37). Their findings showed that SCO reversed a LPS-induced reduction in IκB-α and an increase in the expression of MyD88, NF-κB/p65 and c-Jun proteins.

### PI3K-AKT

The PI3K/AKT/mTOR pathway is thought to be a negative regulator of TLR4/NF-κB signaling pathway in macrophages, and it plays a specific role in the regulation of inflammatory responses (67). In a study performed by Liu et al., treatment with SCO downregulated LPS-induced increases in phosphorylated AKT and mTOR, reduced the accumulation of p62 and LC3 and rescued autophagy via blocking the PI3K/AKT/mTOR pathway in macrophages (15). Treatment with SCO also weakened the phosphorylation of PI3K and AKT in IL-1β-stimulated chondrocytes, suggesting that SCO has a protective effect on IL-1β-induced inflammatory responses in chondrocytes (3).

### Nrf2

Transcription factor Nrf2 plays an important role in the antioxidant and detoxification responses, while its overactivation is harmful (68). Nrf2 is regulated by a high concentration of ROS and affects the transcription of p62 (69). Liu et al. found that SCO reduced Nrf2 protein level induced by the Methionine-Choline deficient (MCD) diet in mice, inhibited the production of a high concentration of ROS and decreased the levels of Nrf2 and phospho-p38 proteins as well as p62 target genes in LPS-stimulated RAW264.7 cells, indicating that SCO alleviates cell damage and inflammation by inhibiting ROS/P38/Nrf2 axis (15).

### JNK/Sab

c-Jun N-terminal kinase (JNK) is a member of the mitogen-activated protein kinase (MAPK) family, and its continuous activation can impair the function of mitochondrial respiratory chains, leading to mitochondrial dysfunction (70). Studies have shown that JNK, when combined with Sab, which is a scaffold protein located in the outer membrane of mitochondria, triggers the destruction of mitochondrial electron transport chain and promotes the release of reactive oxygen species (ROS), ultimately resulting in cell death (71, 72). JNK binds to Sab and separates SHP-1 from Sab, and then the activated SHP-1 is transferred to the mitochondrial intima, thereby causing Src inactivation and mitochondrial dysfunction (73). In a study by Yu et al., it was found that SCO improved PA-induced reduction of mitochondrial membrane potential and ATP production in hepatocytes and downregulated ROS by inhibiting the activation of JNK and SHP-1 and preventing the inactivation of Src (30). Therefore, SCO can restore mitochondrial function by blocking the JNK/Sab activation.

### TGF−β/Smad

TGF-β1, a key regulator of fibrosis in many organs (74), induces expression of NOXs and production of ROS, and is involved in regulating hepatic stellate cells (HSC) activation, which is a key step in initiating liver fibrosis (75). Liu et al. found that administration of SCO suppressed cell proliferation, Smad3 phosphorylation and extracellular matrix (ECM) expression in TGF-β1-induced HSC-T6 cells, accompanied by decreases in the expression of α-SMA, collagen I, collagen III, NOXs and ROS production (41). In addition, Xu et al. also demonstrated that SCO inhibited proliferation, fibrotic phenotype and oxidative stress level of the pancreatic stellate cells by downregulating the expression of α-SMA and type I collagen, and alleviated pancreatic fibrosis through TGF-β/Smad pathway (74). Taken together, SCO can inactivate TGF-β1/Smad3 signaling pathway, therefore improving the pathology and symptoms of fibrosis-related diseases.

### Nitric oxide -cGMP

NO activates cytoplasmic guanylate cyclase, which then increases the level of cGMP in smooth muscle cells, leading to relaxation of smooth muscle cells (76). Choi et al. demonstrated that treatment with SCO increased the level of cGMP in rabbits, resulting in the significant relaxation of their penile corpus cavernosum smooth muscle (PCCSM). This effect of SCO was weakened by blocking NO synthetase or guanylate cyclase using N-ω-nitro-l-arginine methyl ester hydrochloride (L-NAME) or 1H-[1,2,4]oxadiazolo[4,3-a]quinoxalin-1-one (ODQ), suggesting that SCO promotes penile erection by activating the NO-cGMP signaling pathway (77).

### JAK2-STAT3

Janus Kinase 2 (JAK2) can induce the phosphorylation of signal transducer and activator of transcription 3 (STAT3) and is an important regulator of inflammatory responses as well as cell proliferation/differentiation (78). It was found that alterations in JAK2/STAT3 pathway affected the expression of many pro-inflammatory cytokines (79). SCO was reported to suppress the accumulation of STAT3 transported from the cytosol to the nucleus, resulting in the inhibition of vascular smooth muscle cell (VSMC) proliferation through G1 phase arrest and suppression of Rb phosphorylation (80). SCO also exerted antitumor effects on prostate cancer cells by inhibiting the transcription of STAT3 target genes, such as cyclin D1, c-Myc, survivin, Bcl-2 and Socs3, and decreasing the phosphorylation and nuclear accumulation of STAT3, but not JAK2 (81).

## Limitations and future research directions

Currently, SCO is predominantly utilized as a component or ingredient of Artemisiae Scopariae Herba in compound formulations rather than as a standalone treatment. While animal and cellular experiments have provided valuable insights into the effects of SCO on immune cells and immune-based diseases, especially some autoimmune diseases, there is a lack of data concerning its toxicity, pharmacokinetics and side effects. Moreover, there is also a gap between preclinical findings and clinical data since SCO has not been studied in humans. In these aspects, both animal and clinical studies are warranted to ascertain its optimal dosages, efficacy and potential side effects in the treatment of IMIDs, in particular, common autoimmune diseases such as IBD, rheumatoid arthritis, osteoarthritis, lupus and type 1 diabetes. Although SCO holds much promise as an effective therapeutic agent for some IMIDs in preclinical studies, well-designed clinical trials are imperative to provide conclusive evidence and inform clinical practice for treating various autoimmune diseases as well as inflammatory hepatic diseases. Since SCO is not a powerful conventional immunosuppressant, however, it alone may not be sufficient to effectively inhibit inflammation in a clinical setting, which may pose a significant challenge to recruit patients for a large-scale clinical trial. Perhaps, a feasible clinical trial should start to explore the potentially synergistical effects of SCO on IMIDs with other immunomodulatory or anti-inflammatory agents. It’s also imperative to screen its derivatives for a better bioavailability, stability, efficacy or reduced toxicity. We should also carefully observe the potential side effects of SCO in preclinical studies even before clinical trials. Additionally, further studies are required to understand its drug metabolism and pharmacokinetics before the clinical application of SCO to optimize its dosages. Finally, as an additional limitation, previous studies have not identified the exact binding sites of various signaling molecules and pathways. It remains unclear how SCO interacts with each molecule in a specific intracellular signaling axis. Thus, warranted are in-depth studies on exact mechanisms of action underlying its effects, especially its binding sites of various intracellular signaling pathways, such as TLR4/Myd88/NFκB, TGFβR/Smad3 and JNK/Sab/SHP-1 axes.

## Conclusions

Conventional immunosuppressive drugs often carry high costs and may increase the risk of infections and development of cancers. Therefore, it’s imperative to seek alternatives of moderate immunosuppressants with less side effects, such as SCO. Here we have compiled this review showcasing the efficacy of SCO in treating IMIDs, such as allergic rhinitis, osteoarthritis, type 1 diabetes, IBD and neuroinflammatory diseases, and inflammatory hepatic diseases. SCO modulates both adaptive and innate immune cells, including mast cells, monocytes, macrophages, neutrophils and T cells, primarily through regulating their secretion of proinflammatory or anti-inflammatory cytokines. It also has a broad impact on intracellular signal transduction pathways, mainly including TLR4/Myd88/NFκB, TGFβR/Smad3, Nrf2/P38, JAK2/STAT3 and JNK/Sab/SHP-1 etc. Future study should focus on clinical trials to evaluate the toxicity and efficacy of SCO in the treatment of various IMIDs.
